# Case report: A novel loss-of-function pathogenic variant in the KCNA1 cytoplasmic N-terminus causing carbamazepine-responsive type 1 episodic ataxia

**DOI:** 10.3389/fneur.2022.975849

**Published:** 2022-08-09

**Authors:** Rían W. Manville, Richard Sidlow, Geoffrey W. Abbott

**Affiliations:** ^1^Bioelectricity Laboratory, Department of Physiology and Biophysics, School of Medicine, University of California, Irvine, Irvine, CA, United States; ^2^Department of Medical Genetics and Metabolism, Valley Children's Hospital, Madera, CA, United States

**Keywords:** Ataxia, carbamazepine, EA1, *KCNA1*, Kv1.1

## Abstract

Episodic ataxia is an umbrella term for a group of nervous system disorders that adversely and episodically affect movement. Episodes are recurrent, characterized by loss of balance and coordination and can be accompanied by other symptoms ranging from nausea to hemiplegia. Episodic Ataxia Type 1 (EA1) is an inherited, autosomal dominant disease caused by sequence variants in *KCNA1*, which encodes the voltage-gated potassium channel, KCNA1 (Kv1.1). Here we report a novel loss-of-function *KCNA1* pathogenic variant [c.464T>C/p.Leu155Phe] causing frequent, sudden onset of clumsiness or staggering gait in the young female proband. The gene variant was maternally inherited and the mother, whose symptoms also began in childhood, has a normal MRI and EEG, slurred speech and dystonic movements involving upper extremities and mouth. Both mother and daughter are responsive to carbamazepine. Cellular electrophysiology studies of KCNA1-L155P potassium channels revealed complete but non-dominant loss of function, with reduced current and altered gating in heterozygous channels. To our knowledge this is the first EA1-associated pathogenic variant located in the KCNA1 cytoplasmic N-terminus, expanding the reported clinically sensitive domains of the channel.

## Introduction

Ataxias are a group of movement disorders in which affected individuals exhibit loss of balance, loss of coordination, irregular gait and slurred speech. Episodic Ataxia 1 (EA1) is an autosomal dominant inherited form of ataxia caused by genetic variation in the human *KCNA1* gene, which encodes the KCNA1 (Kv1.1) voltage-gated potassium (Kv) channel (1). EA1 patients typically bear one wild-type and one pathogenic variant *KCNA1* allele. EA1 involves altered central and peripheral nerve function, as KCNA1 performs important functions in both the central nervous system and in peripheral nerves (2–4). Centrally, KCNA1 is especially important in the hippocampus, cerebellum, and neocortex; peripherally, KCNA1 is especially critical at synaptic terminal sites and juxtaparanodal segments of the nodes of Ranvier of myelinated axons (5). EA1-associated *KCNA1* mutations generally cause loss of function in KCNA1, most commonly by altering channel functional properties and less commonly by impairing KCNA1 biosynthesis or anterograde trafficking (6, 7).

Episodic ataxia is relatively rare, affecting <1/100,000 people, and is subdivided into at least 7 forms, the most common being EA1 and EA2. EA2 is caused by pathogenic variants in *CACNA1A*, which encodes the α1A pore-forming subunit of the Cav1.2 neuronal voltage-gated P/Q-type calcium channel (8). Episodic ataxia symptoms include recurring episodes of poor coordination and balance, and in addition can comprise blurred vision, slurred speech, vertigo, nausea and emesis, migraines, tinnitus, muscle weakness, hemiplegia, seizures, and myokymia (predominantly in the interictal interval in EA1) (9, 10). EA1 and EA2 are typically treated with anticonvulsant/antiseizure medications such as carbamazepine, valproic acid and acetazolamide, although the latter is generally more effective at treating EA2 (11, 12).

## Case presentation

A 13-year-old female (the proband) was first seen by a geneticist in 2019 for the chief complaint of a personal and family history of episodic abnormal movements involving sudden onset of clumsiness or staggering gait without any alteration in sensorium, lasting 30 s-2 min. Her neurological symptoms began at age two and she was first seen by a Valley Children's Hospital neurologist at age four, but was not genetically evaluated at that time. The longer the episode, the greater the likelihood that slurring of speech will occur in the proband. Her episodes can be triggered by heightened activity or by fatigue, but neither is necessary for an episode to occur, and the proband averages 1–2 episodes per week, responsive to carbamazepine. The proband's gestation, birth and developmental history are normal and there is no other medical or surgical history other than familial short stature. The short stature arises from a familial delayed growth pattern of unknown mechanism although it is possible there is transient growth hormone resistance.

The proband's physical exam and neurological exam were normal. No MRI was performed on the proband since the clinical impression was episodic ataxia - and she was responsive to the medication prescribed for this (carbamazepine) - based on family history. Specifically, the proband's mother began presenting with neurological symptoms at age 8, which included slurring of speech, ataxia, and dystonic movements involving her upper extremities and mouth. MRI and EEG were performed on the proband's mother when she was 8 years old and were reported as negative, yet her episodes were initially diagnosed as epilepsy. Later, upon presentation of the proband, a diagnosis of ataxia was proposed for mother and daughter, and genetic analyses conducted based on this.

The proband's EEG was read as negative by the same neurologist that has been following the proband for 11 years. Specifically, at 3 years of age, the proband underwent measurements with a 21-channel digital EEG machine with ECG, respiration and eye movement monitors using the International 10–20-electrode placement system. The EEG was collected using 28 leads including ECG, EOG and respiration artifact leads. During the record the patient was at various times awake, drowsy and asleep. At the onset of the record, the patient was awake and displayed a normal anterior-posterior gradient with faster rhythms anteriorly. Maximum posterior dominant rhythm was up to 9 Hz and was symmetric. Movement artifact was very prominent as well as muscle artifact during the awake portion of the record. Photic stimulation and hyperventilation were not performed during the study. As the patient fell into drowsiness and sleep, symmetric sleep spindles and vertex sharp waves were observed. At times the vertex sharp waves occurred in runs, but there were no focal asymmetries or epileptiform features seen during the awake portion or during sleep. Upon arousal, a normal awake background returned. Thus, the neurologist reported a normal awake and sleep EEG.

**Figure 1:**
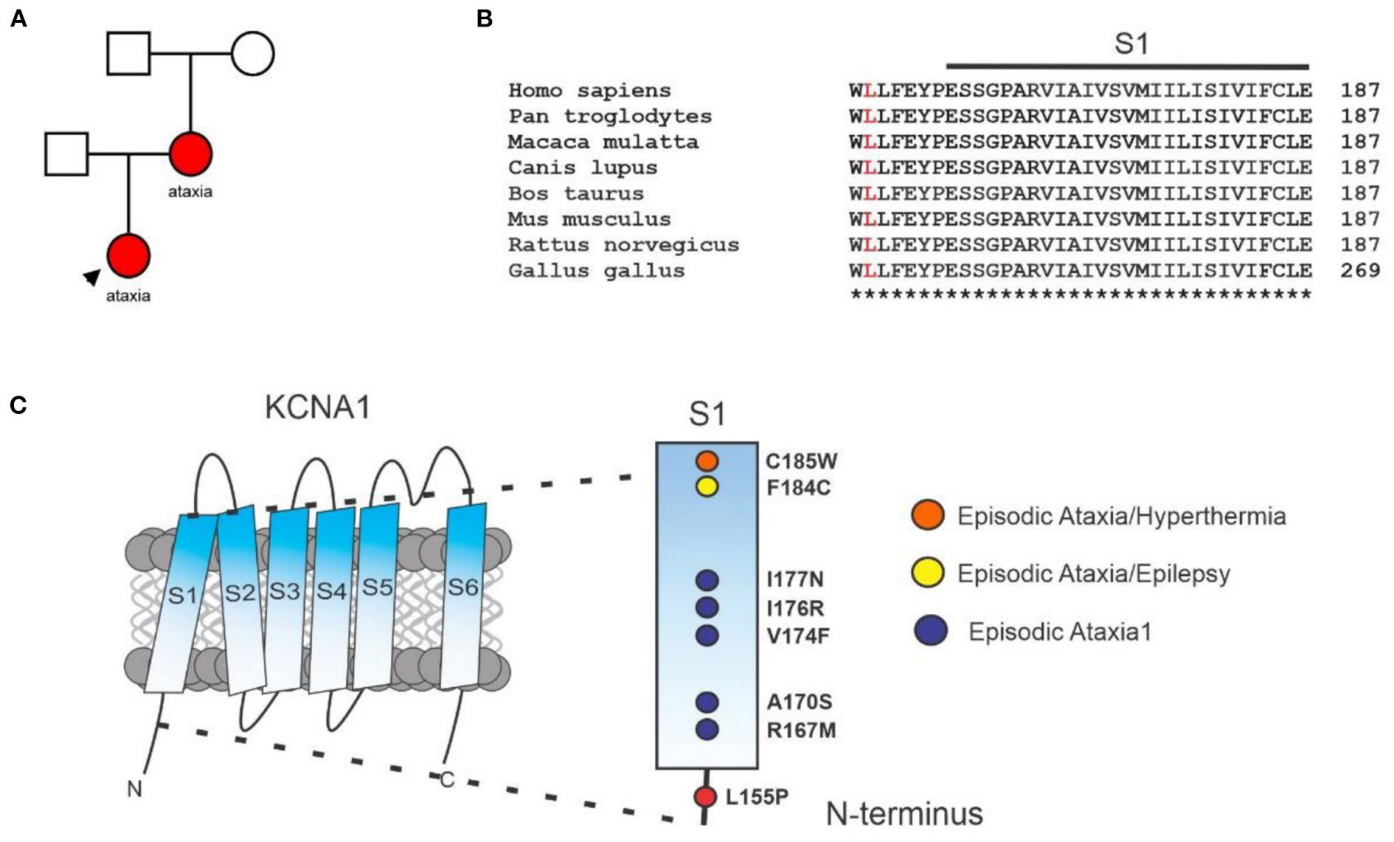
Clinical genetics. **(A)** Pedigree diagram for KCNA1-L155P (red) in the family under study. **(B)** Sequence alignment for S1 and S1-proximal N-terminal segment of KCNA1 with L155 highlighted (red). **(C)** Location of L155P and some previously reported EA1 sequence variants in KCNA1 (see Discussion).

The initial karyotype and limited channelopathy/hereditary ataxia panels (*PRRT2, SLC2A1, ATP1A2*, and *ATP1A3*) of the proband were negative. More recently, an expanded ataxia panel (GeneDx.com) was conducted using next-generation sequencing with copy number variant (CNV) detection (Supplementary Data 1). The sequencing revealed a gene variant in the coding region of *KCNA1*: c.464 T>C, encoding KCNA1: p.(Leu155Phe). The proband was heterozygous for p.(Leu155Phe); 100% of the *KCNA1* coding region was covered at a minimum of 10x and there was no indication of a multi-exon deletion or duplication. The proband's mother was also found by next generation sequencing to be heterozygous for the p.(Leu155Phe) variant in *KCNA1*, while the proband's father does not harbor the variant. The mutation is apparently *de novo* in the proband's mother, as the maternal grandmother and grandfather of the proband were tested and were negative (Figure 1A). KCNA1-L155 lies in the S1-proximal portion of the cytoplasmic N-terminus, is highly conserved (Figure 1B), and is close to other EA1-linked sequence variants (Figure 1C).

## Functional characterization of KCNA1-L155P potassium channels

KCNA1-L55P is not observed at significant frequency in large population cohorts (The Genome Aggregation Database; gnomAD) and has not to our knowledge been previously reported as either benign or pathogenic (13). KCNA1-L155P cDNA was generated (Genscript, Piscataway, NJ) in the pTLNx expression vector, and then from this we generated cRNA by *in vitro* transcription with the mMessage mMachine SP6 kit (ThermoFisher, Waltham, MA). We injected wild-type KCNA1 (2 ng), KCNA1-L155P cRNA (2 ng) or wild-type KCNA1 + KCNA1-L155P (A1/A1-L155P) cRNA (2 ng each) into stage V and VI defolliculated *Xenopus laevis* oocytes. Oocytes were incubated at 16 °C for 2 days and then currents recorded using two-electrode voltage-clamp. Voltage protocols are shown in the figures.

**Figure 2:**
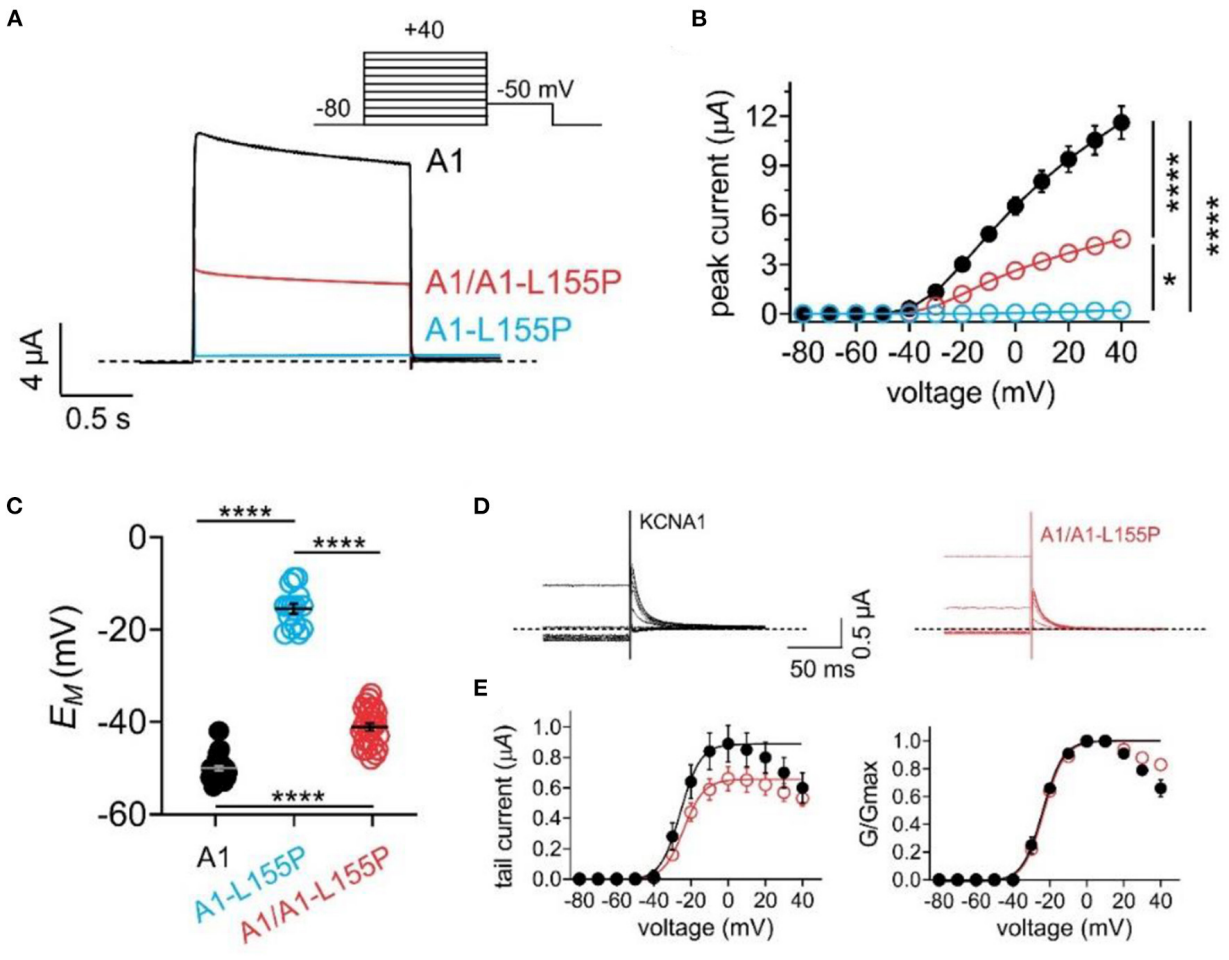
Effects of L155P on KCNA1 activity. Error bars indicate SEM. *n* indicates number of oocytes. Statistical comparisons by one-way ANOVA; *P < 0.05; ****P < 0.0001. Dashed line indicates zero current level. **(A)** Mean traces for wild-type (A1), homozygous mutant (A1-L155P) and heterozygous mutant (A1/A1-L155P) channels expressed in oocytes. Scale bars lower left inset; voltage protocol upper inset; *n* = 15–29 per group (see Table 1 for values). **(B)** Mean peak current for traces as in **(A)**; *n* = 15–29 per group (see Table 1). **(C)** Mean unclamped oocyte membrane potential for oocytes as in **(A)**; *n* = 15–29 per group (see Table 1 for values). **(D)** Inset showing tail currents at −50 mV after prepulses similar to those shown in **(A)**. **(E)** Mean peak tail current (left) and normalized tail current (right) for currents as in **(D)** (*n* = 26–29) (see Table 1 for values).

As expected, wild-type KCNA1 channels generated robust, voltage-dependent outward currents in response to depolarizing voltage pulses. In contrast, homomeric KCNA1-L155P channels were nonfunctional. Currents generated by equal co-injection of wild-type and L155P KCNA1 (A1/A1-L155P) generated currents with 39% of the peak current magnitude of wild-type KCNA1 (Figures 2A,B, Table 1). Reflecting their inability to generate outward K^+^ current at resting membrane potential in oocytes, the mean resting membrane potential (*E*_M_) of unclamped oocytes expressing homomeric KCNA1-L155P was 34 mV more positive than oocytes expressing homomeric wild-type KCNA1, while the mean resting membrane potential of oocytes expressing A1/A1-L155P channels was 8 mV more positive than oocytes expressing homomeric wild-type KCNA1 (Figure 2C, Table 1). The shift in membrane potential for oocytes expressing A1/A1-L155P channels was attributable to voltage-independent reduction in current magnitude; there was no quantifiable difference in the voltage dependence of A1/A1-L155P compared to wild-type KCNA1 (Figures 2D,E).

**Table 1:** Cellular electrophysiological characteristics of wild-type (WT) KCNA1, and homozygous and heterozygous KCNA1-L155P potassium channels.

	**Peak current density +40 mV (μA)**	**Non-normalized V_0.5_ (mV)**	***EM* (mV)**	**Inactivation V_0.5_ (mV)**
KCNA1	11.61 ± 0.9 (*n* = 29)	−25.41 ± 1.9 (*n* = 29)	−49.5 ± 0.5 (*n* = 26)	−37.9 ± 0.8 (*n* = 16)
KCNA1-L155P	0.21 ± 0.02 (*n* = 15)	*n.a*	−15.6 ± 1.1 (*n* = 15)	*n.a*
KCNA1/KCNA1-L155P	4.53 ± 0.5 (*n* = 26)	−23.73 ± 1.4 (*n* = 26)	−41.2 ± 0.7 (*n* = 26)	−46.3 ± 0.7 (*n* = 12)

*n.a., not applicable*.

**Figure 3:**
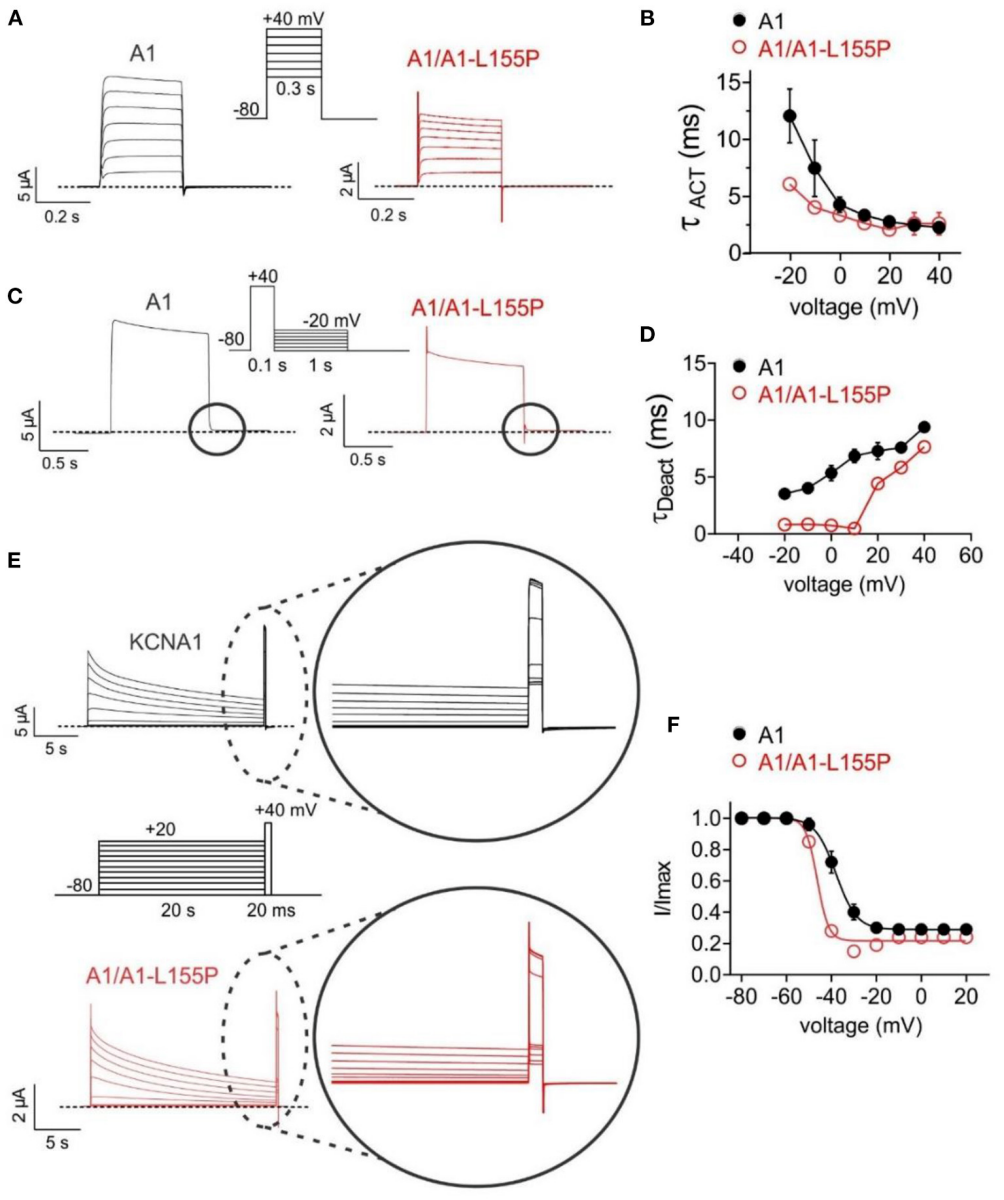
Effects of L155P on KCNA1 gating. Error bars indicate SEM. *n* indicates number of oocytes. Dashed line indicates zero current level. **(A)** Mean traces for wild-type (A1) and heterozygous mutant (A1/A1-L155P) channels expressed in oocytes using the voltage protocol shown (upper inset). Scale bars lower left inset; *n* = 12–16 per group. **(B)** Mean activation rate (T_ACT_) calculated using a single exponential function from traces as in **(A)**; *n* = 12–16 per group. **(C)** Mean traces for wild-type (A1) and heterozygous mutant (A1/A1-L155P) channels expressed in oocytes using the voltage protocol shown (upper inset). Scale bars lower left inset; *n* = 12–16 per group. **(D)** Mean deactivation rate (T_Deact_) calculated using a single exponential function from the circled portion of traces as in **(C)**; *n* = 12–16 per group. **(E)** Mean traces for wild-type (A1) and heterozygous mutant (A1/A1-L155P) channels expressed in oocytes using the voltage protocol shown (center inset). Scale bars lower left inset; *n* = 12–16 per group. **(F)** Mean proportion of remaining non-inactivated current calculated from the circled portion of traces as in **(E)**; *n* = 12–16 per group (see Table 1 for values).

Compared to wild-type KCNA1 channels, A1/A1-L155P channels exhibited twofold faster activation (Figures 3A,B) and several-fold faster deactivation (depending on voltage) (Figures 3C,D). KCNA1 channels also exhibit voltage-dependent inactivation following activation; compared to wild-type, A1/A1-L155P exhibited negative-shifted voltage dependence of inactivation such that there was, e.g., a threefold greater proportion of A1/A1-L155P channels inactivated at −40 mV compared to wild-type KCNA1, and a −8.4 mV shift in the voltage dependence of inactivation (Figures 3E,F, Table 1).

## Discussion

KCNA1 channel activity generally dampens neuronal excitability; therefore, *KCNA1* loss-of-function mutations such as those in EA1 increase neuronal excitability, lowering the threshold for action potential generation. This in turn can result in increased firing frequency, broadening of individual action potentials, and an increase in neurotransmitter release (14). As KCNA1 channels form homomeric and heteromeric Kv channel complexes (e.g., with KCNA2) at juxtaparanodal regions and branch points of myelinated axons, their normal function is needed for healthy neuromuscular transmission and to limit aberrant axonal firing. These processes are disrupted when KCNA1 current is pharmacologically blocked or impaired by loss-of-function mutations (5, 15, 16), such as the newly discovered L155P variant.

KCNA1 channels generated from 50/50 wild-type and L155P cRNA to mimic the heterozygous proband and her mother described here, show 61% reduced peak current and oocytes expressing them exhibit +8 mV-depolarized resting membrane potential, i.e., increased excitability, compared to oocytes expressing wild-type KCNA1 alone. Moreover, A1/A1-L155P channel deactivated several-fold faster at suprathreshold potentials (Figure 3D), which could prolong action potentials or decrease the time between action potentials. In addition, A1/A1-L155P currents inactivated more completely than those of wild-type KCNA1, especially around −20 to −40 mV, where the consequently reduced current could impair neuronal repolarization (Figure 3F).

The altered functional properties of homomeric L155P and heteromeric A1/A1-L155P channels suggest that L155P subunits are able to co-assemble with wild-type KCNA1 and alter its properties. L155P effects are not dominant negative as otherwise, using a binomial distribution and tetrameric stoichiometry of KCNA1 channels, L155P would reduce peak current to 1/16 that of wild-type KCNA1. To our knowledge, L155P is the first reported EA1-associated mutation to be located in the cytoplasmic N-terminus of KCNA1. The EA1 pathogenic variants previously found that locate closest to L155P lie in KCNA1 S1, i.e., the first transmembrane segment (Figure 1C). Of these, R167M, A170S, V174F, I176R, I177N cause “pure” EA1; further up S1 and closer to its extracellular end lie F184C, linked to both EA1 and epilepsy, and C185W, linked to EA1 and hyperthermia. The other tight clustering of “pure” EA1 variants lies in the intracellular end of S4 (F303V, L305F, R307C) and intracellular S4-5 linker (G311D/S, I314T); throughout the remainder of the channel, pure EA1 and mixed-phenotype KCNA1-associated disorders are mingled (Supplementary Table 1) (13).

Future studies on L155P outside the scope of this case study will include an examination of its behavior in heteromeric complexes with KCNA2 and KCNA4, with which KCNA1 is thought to co-assemble *in vivo* in addition to forming homomeric KCNA1 channels as it is possible this may give some clues to why some KCNA1 mutants cause expanded phenotypes in addition to episodic ataxia (17–19). However, EA1 is notoriously variable in its phenotypes (20), with even identical twins exhibiting different degrees of ataxia severity (21). Some EA1 symptoms also overlap with those of epilepsy, which can lead to misdiagnosis. In the current case, the proband's mother was initially misdiagnosed with epilepsy based on overt neurological symptoms (but no seizures), despite her EEG and MRI being normal. The case emphasizes the importance of considering episodic ataxia as an alternate diagnosis to epilepsy under these circumstances. This first report of a KCNA1 variant in the N-terminus being associated with EA1, and in the absence of other EA1-linked disorders, expands the cluster of “pure” EA1 sequence variants near the intracellular end of S1 (Figure 1C, Supplementary Table 1).

## Data availability statement

The original contributions presented in the study are included in the article/Supplementary material, further inquiries can be directed to the corresponding author.

## Ethics statement

Written informed consent was obtained from the individual(s), and minor(s)' legal guardian/next of kin, for the publication of any potentially identifiable images or data included in this article.

## Author contributions

RS contributed clinical and human gene sequencing data and analysis. RM conducted cellular electrophysiology studies and analysis. GA prepared the original manuscript draft. All authors contributed to the intellectual content, edited the manuscript, and approved the submitted version.

## Funding

This study was supported by National Institute of Neurological Disorders and Stroke, National Institutes of Health (NS107671 to GA). The funding body did not play a role in the data collection, analysis, or interpretation, neither did it have input to the manuscript content.

## Conflict of interest

The authors declare that the research was conducted in the absence of any commercial or financial relationships that could be construed as a potential conflict of interest.

## Publisher's note

All claims expressed in this article are solely those of the authors and do not necessarily represent those of their affiliated organizations, or those of the publisher, the editors and the reviewers. Any product that may be evaluated in this article, or claim that may be made by its manufacturer, is not guaranteed or endorsed by the publisher.
