# Supplementary material for: Case report: A novel loss-of-function pathogenic variant in the KCNA1 cytoplasmic N-terminus causing carbamazepine-responsive type 1 episodic ataxia
Source: Front Neurol. 2022 Aug 9;13:975849. doi: 10.3389/fneur.2022.975849 (PMC9397541; doi:10.3389/fneur.2022.975849)
Supplement: Supplementary file 1 [file Data_Sheet_1.PDF]

Supplementary Data 1 – Expanded Ataxia Genetics Panel Gene Coverage

The following genes were specifically reviewed, with the percentage of the coding region covered with more than 10 reads (10X) indicated in parentheses: AAAS (100%), AARS (100%), AARS2 (100%), AASS (100%), ABCA1 (100%), ABCA5 (100%), ABCA7 (100%), ABCB7 (100%), ABCC6 (93.26%), ABCC8 (100%), ABCD1 (100%), ABHD12 (100%), ABHD5 (100%), ACAT2 (100%), ACBD5 (100%), ACBD6 (100%), ACD (100%), ACO2 (100%), ACOX1 (100%), ACOX2 (100%), ACP2 (100%), ACP5 (100%), ACTA1 (100%), ACTB (100%), ADAR (100%), ADCK3 (100%), ADCY5 (100%), ADCY6 (100%), ADD3 (100%), ADGRG1 (100%), ADK (100%), ADPRHL2 (100%), ADRA2B (100%), ADSL (100%), AFF2 (100%), AFG3L2 (100%), AGA (100%), AGRN (100%), AGTPBP1 (100%), AHI1 (100%), AIFM1 (100%), AIMP1 (100%), AIMP2 (100%), AIP (100%), AIPL1 (100%), AKT1 (100%), ALAD (100%), ALDH18A1 (100%), ALDH3A2 (100%), ALDH5A1 (100%), ALDH6A1 (100%), ALDH7A1 (100%), ALG11 (100%), ALG6 (100%), ALK (100%), ALS2 (100%), AMACR (100%), AMPD2 (100%), ANG (100%), ANGPTL6 (100%), ANK3 (100%), ANKLE2 (100%), ANKRD11 (100%), ANO10 (100%), ANO3 (100%), ANOS1 (100%), ANXA11 (100%), AP1S2 (100%), AP3B2 (100%), AP3D1 (98.8%), AP4B1 (100%), AP4E1 (100%), AP4M1 (100%), AP4S1 (100%), AP5Z1 (100%), APOA1 (100%), APOA1BP (100%), APOB (99.99%), APOPT1 (100%), APP (100%), APTX (100%), ARCN1 (100%), ARFGEF2 (100%), ARG1 (100%), ARHGAP31 (100%), ARID1B (100%), ARL13B (100%), ARL6 (100%), ARL6IP1 (100%), ARMC9 (100%), ARNT2 (100%), ARSA (100%), ARSI (100%), ARV1 (100%), ARX (100%), ASL (100%), ASNS (100%), ASPA (100%), ASPM (100%), ASS1 (100%), ATAD3A (100%), ATAD3B (100%), ATCAY (100%), ATF4 (100%), ATG5 (100%), ATL1 (100%), ATM (100%), ATP13A2 (100%), ATP1A2 (100%), ATP1A3 (100%), ATP2B3 (100%), ATP2B4 (100%), ATP5A1 (100%), ATP5E (100%), ATP6AP2 (100%), ATP6V0A2 (100%), ATP6V1A (100%), ATP6V1E1 (100%), ATP7A (100%), ATP7B (100%), ATP8A2 (100%), ATPAF1 (100%), ATPAF2 (100%), ATRX (100%), AUH (100%), B3GALNT2 (100%), B3GALT6 (100%), B4GALNT1 (100%), B4GAT1 (100%), B9D1 (100%), BAZ1B (100%), BBIP1 (100%), BBS1 (100%), BBS10 (100%), BBS12 (100%), BBS2 (100%), BBS4 (100%), BBS5 (100%), BBS7 (100%), BBS9 (100%), BCAP31 (100%), BCKDHA (100%), BCKDHB (100%), BCL11B (100%), BCOR (100%), BCS1L (100%), BICD2 (100%), BMP15 (100%), BOLA3 (100%), BRAT1 (100%), BSCL2 (100%), BTD (100%), C10orf2 (100%), C11orf73 (100%), C12orf65 (100%), C19orf12 (94.81%), C19orf70 (100%), C21orf2 (100%), C5orf42 (100%), CA8 (100%), CACNA1A (100%), CACNA1B (100%), CACNA1D (100%), CACNA1G (100%), CACNB4 (100%), CACNG2 (100%), CAMTA1 (100%), CAMTA2 (100%), CAPN1 (100%), CARS2 (100%), CASK (100%), CASR (100%), CAV1 (100%), CBS (100%), CC2D1A (100%), CC2D2A (100%), CCDC141 (100%), CCDC82 (100%), CCDC88C (100%), CCNF (100%), CCR1 (100%), CCT5 (100%), CDC42 (100%), CDH15 (100%), CDH23 (100%), CDK5RAP2 (100%), CDK6 (100%), CDKL5 (97.27%), CECR1 (100%), CENPJ (100%), CEP104 (100%), CEP120 (100%), CEP135 (100%), CEP152 (89.55%), CEP164 (100%), CEP290 (100%), CEP41 (100%), CEP63 (100%), CEP78 (100%), CFL2 (100%), CHAMP1 (100%), CHAT (100%), CHCHD10 (100%), CHD2 (100%), CHD7 (100%), CHL1 (100%), CHMP1A (100%), CHMP2B (100%), CHN1 (100%), CHRNA4 (100%), CHRNA7 (100%), CIB2 (100%), CISD2 (100%), CIT (100%), CIZ1 (97.31%), CKAP2L (100%), CLCN2 (100%), CLIC2 (100%), CLIP1 (100%), CLIP2 (100%), CLK2 (100%), CLMP (100%), CLN3 (100%), CLN5 (100%), CLN6 (100%), CLN8 (100%), CLP1 (100%), CLPB (100%), CLPP (100%), CLRN1 (100%), CNTN4 (100%), CNTNAP1 (100%), COA3 (100%), COA5 (100%), COA7 (100%), COASY (100%), COG2 (100%), COG4 (100%), COG5 (100%), COL12A1 (100%), COL13A1 (99.64%), COL18A1 (99.7%), COL25A1 (100%), COL4A1 (100%), COL4A2 (100%), COL6A1 (100%), COL6A2 (100%), COL6A3 (100%), COLEC11 (100%), COQ2 (100%), COQ5 (100%), COQ7 (100%), COQ9 (100%), COX10 (100%), COX14 (100%), COX15 (94.3%), COX20 (100%), COX6B1 (100%), CP (100%), CPS1 (100%), CPT1A (100%), CPT1C (100%), CRADD (100%), CRAT (100%), CRB1 (100%), CRBN (100%), CREBBP (100%), CRLF1 (99.69%), CRX (100%), CSF1R (100%), CSPP1 (100%), CSTB (100%), CTBP1 (100%), CTC1 (100%), CTDP1 (100%), CTLA4 (100%), CTNNB1 (100%), CTNND2 (100%), CTSD (100%), CTSF (100%), CUL4B (100%), CWF19L1 (100%), CYB5R3 (100%), CYC1 (100%), CYP26C1 (100%), CYP27A1 (100%), CYP2U1 (100%), CYP7B1 (100%), DAB1 (100%), DAG1 (100%), DAO (100%), DARS (85.48%), DARS2 (100%), DBT (100%), DCAF17 (100%), DCC (100%), DCTN1 (100%), DCX (100%), DDB2 (100%), DDC (100%), DDHD1 (100%), DDHD2 (100%), DDX3X (97.55%), DEAF1 (100%), DFNB31 (100%), DHCR24 (100%), DHDDS (100%), DHFR (100%), DHH (100%), DKC1 (100%), DKK1 (100%), DLAT (100%), DLD (100%), DLG3 (100%), DLGAP4 (100%), DLL4 (100%), DMXL2 (100%), DNA2 (100%), DNAJC12 (100%), DNAJC13 (100%), DNAJC19 (100%), DNAJC3 (100%), DNAJC5 (100%), DNAJC6 (100%), DNAL4 (100%), DNASE1L3 (100%), DNM1 (100%), DNM1L (100%), DNM2 (100%), DNMT1 (100%), DOCK3 (100%), DOCK6 (100%), DOCK8 (100%), DPM1 (100%), DPYD (100%), DPYS (100%), DRD2 (100%), DSTYK (100%), DUSP6 (100%), DYNC1H1 (100%), DYRK1A (100%), DYSF (100%), EARS2 (100%), EBF3 (100%), ECHS1 (100%), ECM1 (100%), EDC3 (100%), EDN3 (100%), EDNRB (100%), EED (100%), EEF2 (100%), EGR2 (100%), EHMT1 (100%), EIF2B1 (100%), EIF2B2 (100%), EIF2B3 (100%), EIF2B4 (100%), EIF2B5 (100%), EIF2S3 (100%), EIF4G1 (100%), ELF2 (100%), ELN (100%), ELOVL4 (100%), ELOVL5 (100%), ELP2 (100%), EMC1 (100%), EML1 (100%), EMX2 (100%), ENG (100%), ENPP1 (100%), ENTPD1 (100%), EOGT (100%), EP300 (100%), EPB41L1 (100%), EPB41L4A (100%), EPCAM (100%), EPHA4 (100%), EPT1 (100%), ERAP1 (98.95%), ERBB4 (100%), ERCC1 (100%), ERCC2 (100%), ERCC3 (100%), ERCC4 (100%), ERCC5 (100%), ERCC6 (100%), ERCC8 (100%), ERLIN1 (100%), ERLIN2 (100%), ERMARD (100%), ETHE1 (100%), EXOSC3 (100%), EXOSC8 (100%), EXOSC9 (96.24%), EXT1 (100%), EXT2 (100%), EZH2 (100%), FA2H (100%), FAAH (100%), FAM134B (100%), FAN1 (100%), FAR1 (100%), FARS2 (100%), FAS (100%), FASTKD2 (100%), FAT1 (100%), FAT2 (100%), FBXL4 (100%), FBXO31 (100%), FBXO7 (100%), FDXR (100%), FEZF1 (100%), FGD4 (100%), FGF12 (100%), FGF14 (100%), FGF17 (100%), FGF8 (100%), FGFR1 (100%), FGFR2 (100%), FGFR3 (100%), FIG4 (100%), FITM2 (100%), FKRP (100%), FKTN (100%), FLNA (100%), FLRT1 (100%), FLRT3 (100%), FLVCR1 (100%), FMN2 (99.89%), FOXG1 (100%), FOXI1 (100%), FOXP1 (100%), FOXP3 (100%), FOXRED1 (100%), FRMD4A (100%), FRRS1L (100%), FSHR (100%), FTL (100%), FUCA1 (100%), FUS (100%), FUZ (100%), FXN (100%), GABBR2 (100%), GABRA1 (100%), GABRA3 (100%), GABRB1 (100%), GABRB2 (92.58%), GABRB3 (100%), GABRD (100%), GABRG2 (100%), GAD1 (100%), GALC (100%), GALT (100%), GAMT (100%), GAN (100%), GATAD2B (100%), GBA (100%), GBA2 (100%), GBE1 (100%), GCDH (100%), GCH1 (100%), GCK (100%), GDF3 (100%), GDF6 (100%), GEMIN4 (100%), GEMIN5 (100%), GFAP (100%), GFM1 (100%), GFM2 (100%), GIGYF2 (99.93%), GJA1 (100%), GJB1 (100%), GJB2 (100%), GJB6 (100%), GJC2 (100%), GLB1 (100%), GLE1 (100%), GLI2 (100%), GLRA1 (100%), GLRB (100%), GLRX5 (100%), GLYCTK (100%), GM2A (100%), GMPPA (100%), GMPPB (100%), GNA11 (100%), GNAL (100%), GNAO1 (100%), GNAQ (100%), GNAS (100%), GNB1 (100%), GNRH1 (100%), GNRHR (100%), GOSR2 (100%), GPHN (100%), GPI (100%), GPR101 (100%), GPR88 (100%), GPR98 (100%), GRID2 (100%), GRIK2 (100%), GRIN1 (100%), GRIN2B (100%), GRM1 (100%), GRN (100%), GSS (100%), GTF2I (58.55%), GTF2IRD1 (100%), GUCY1A3 (100%), GUCY2D (100%), GUF1 (100%), HACE1 (99.31%), HARS (100%), HARS2 (100%), HCFC1 (100%), HCN1 (100%), HDAC8 (100%), HEPACAM (100%), HERC1 (100%), HESX1 (100%), HEXA (100%), HEXB (100%), HFE (100%), HGSNAT (100%), HIBCH (100%), HIC1 (100%), HK1 (100%), HLA-DPB1 (100%), HLCS (100%), HMBS (100%), HMGCS2 (100%), HNRNPA1 (100%), HNRNPA2B1 (100%), HNRNPH2 (100%), HPCA (100%), HPDL (100%), HPGD (100%), HPRT1 (100%), HS6ST1 (100%), HSD17B10 (100%), HSD17B4 (100%), HSPD1 (100%), HSPG2 (99.96%), HTRA1 (100%), HTRA2 (100%), HYLS1 (100%), IARS (100%), IBA57 (100%), IDH2 (100%), IDUA (100%), IFIH1 (100%), IFRD1 (99.49%), IFT140 (100%), IFT172 (100%), IFT27 (100%), IKBKAP (100%), IL10 (100%), IL12A (100%), IL17RD (100%), IL23R (100%), IL6 (100%), IMPDH1 (100%), INPP5E (100%), INPP5K (100%), INS (100%), INVS (100%), IQCB1 (100%), IQSEC2 (98.43%), ISCA2 (100%), ISPD (100%), ITM2B (100%), ITPR1 (100%), JAM3 (100%), KANK1 (100%), KAT6A (100%), KATNB1 (100%), KCNA1 (100%), KCNA2 (100%), KCNAB2 (100%), KCNB1 (100%), KCNB2 (100%), KCNC1 (100%), KCNC3 (90.99%), KCND3 (100%), KCNJ10 (100%), KCNJ11 (100%), KCNJ13 (100%), KCNJ18 (100%), KCNJ6 (100%), KCNMA1 (100%), KCNQ2 (100%), KCNQ3 (100%), KCNT1 (100%), KCTD17 (100%), KCTD7 (100%), KDM5C (100%), KDM6A (100%), KIAA0196 (100%), KIAA0226 (100%), KIAA0556 (100%), KIAA0586 (95.77%), KIAA0753 (100%), KIAA1033 (100%), KIAA2022 (100%), KIDINS220 (100%), KIF11 (100%), KIF1A (100%), KIF1C (100%), KIF26B (100%), KIF2A (100%), KIF5A (100%), KIF5C (10.14%), KIF7 (100%), KIRREL3 (100%), KISS1 (100%), KISS1R (100%), KIT (100%), KLC2 (100%), KLC4 (100%), KLHL40 (100%), KLHL41 (100%), KLLN (100%), KLRC4 (100%), KMT2A (100%), KMT2B (99.71%), KRAS (100%), KY (100%), L1CAM (100%), L2HGDH (100%), LAGE3 (100%), LAMA1 (100%), LAMA2 (100%), LAMB1 (100%), LAMB2 (100%), LARGE (100%), LARS (100%), LARS2 (100%), LCA5 (100%), LDLR (100%), LETM1 (100%), LHX4 (100%), LIAS (100%), LIMK1 (100%), LIMS2 (100%), LIPT1 (100%), LMBRD1 (100%), LMNB1 (100%), LMNB2 (100%), LMOD3 (100%), LRAT (100%), LRPPRC (100%), LRRK2 (100%), LRSAM1 (100%), LYRM7 (100%), LYST (100%), LZTFL1 (100%), MAFB (100%), MAG (100%), MAN1B1 (100%), MAN2B1 (100%), MAPT (100%), MARS (100%), MARS2 (100%), MAST1 (100%), MAT1A (100%), MATR3 (100%), MBD5 (100%), MCCC1 (100%), MCCC2 (100%), MCOLN1 (100%), MCPH1 (100%), MECP2 (100%), MECR (100%), MED13L (100%), MED17 (100%), MED23 (100%), MED25 (100%), MED27 (100%),

MEF2C (100%), MEFV (100%), MEOX1 (100%), METTL23 (100%), MFF (100%), MFN2 (100%), MFSD2A (100%), MFSD8 (100%), MGME1 (100%), MICU1 (100%), MICU2 (100%), MKKS (100%), MKS1 (100%), MLC1 (100%), MLH3 (100%), MLLT3 (100%), MMADHC (100%), MME (100%), MOCS1 (100%), MOCS2 (100%), MPDU1 (100%), MPP7 (100%), MPV17 (100%), MPZ (100%), MR1 (100%), MRE11A (100%), MRPS22 (100%), MSTO1 (97.86%), MTFMT (100%), MTO1 (100%), MTOR (100%), MTPAP (100%), MTTP (100%), MUT (100%), MVK (100%), MYCN (100%), MYD88 (100%), MYH2 (100%), MYO5A (100%), MYO7A (100%), MYO9A (100%), MYT1L (100%), NAA10 (100%), NACC1 (100%), NADK2 (100%), NAGA (100%), NAGLU (100%), NAGPA (100%), NALCN (100%), NANS (100%), NARS2 (100%), NAT8L (100%), NDE1 (100%), NDRG1 (100%), NDST1 (100%), NDUFA1 (100%), NDUFA10 (100%), NDUFA11 (100%), NDUFA12 (100%), NDUFA13 (100%), NDUFA2 (100%), NDUFA4 (100%), NDUFA9 (100%), NDUFAF1 (100%), NDUFAF2 (100%), NDUFAF3 (100%), NDUFAF4 (100%), NDUFAF5 (100%), NDUFAF6 (100%), NDUFB3 (100%), NDUFB9 (100%), NDUFS1 (100%), NDUFS2 (100%), NDUFS3 (100%), NDUFS4 (100%), NDUFS6 (100%), NDUFS7 (100%), NDUFS8 (100%), NDUFV1 (100%), NDUFV2 (100%), NEB (91.7%), NECAP1 (100%), NEFH (100%), NEFL (99.94%), NEK1 (100%), NELFA (100%), NEU1 (100%), NF1 (100%), NFU1 (100%), NGLY1 (100%), NHP2 (100%), NIPA1 (100%), NKAIN2 (100%), NKX2-1 (100%), NKX6-2 (98.59%), NME1 (100%), NMNAT1 (100%), NOD2 (100%), NOL3 (100%), NONO (100%), NOP10 (100%), NOTCH1 (100%), NOTCH3 (100%), NPC1 (100%), NPC2 (100%), NPHP1 (100%), NPHP3 (100%), NPHP4 (100%), NR4A2 (100%), NR5A1 (100%), NRAS (100%), NRD1 (100%), NSD1 (100%), NSMF (100%), NSUN2 (100%), NT5C2 (100%), NTN1 (100%), NTNG1 (100%), NUBPL (100%), NUP107 (100%), NUP62 (100%), NUS1 (100%), OBFC1 (100%), OCLN (92.64%), OFD1 (100%), OGDH (100%), OPA1 (100%), OPA3 (100%), OPHN1 (100%), OPTN (100%), OSGEP (100%), OTC (100%), OTUD4 (100%), OTUD6B (100%), OTX2 (100%), PACRG (86.85%), PAFAH1B1 (100%), PAFAH1B3 (100%), PAH (100%), PAK3 (100%), PANK2 (100%), PARK2 (100%), PARK7 (100%), PARN (100%), PAX3 (100%), PAX6 (100%), PCCA (100%), PCCB (100%), PCDH12 (100%), PCDH15 (100%), PCDH19 (100%), PCLO (99.77%), PCNA (100%), PCYT1A (100%), PDC (100%), PDE10A (100%), PDE2A (100%), PDE6D (100%), PDE8B (100%), PDGFB (100%), PDGFRB (100%), PDHA1 (100%), PDHX (100%), PDP1 (100%), PDSS2 (100%), PDX1 (100%), PDYN (100%), PDZD7 (100%), PET100 (100%), PEX1 (100%), PEX10 (100%), PEX11B (100%), PEX12 (100%), PEX13 (100%), PEX14 (100%), PEX16 (100%), PEX19 (100%), PEX2 (100%), PEX26 (100%), PEX3 (100%), PEX5 (100%), PEX6 (100%), PEX7 (100%), PFN1 (100%), PGAP1 (99.86%), PGK1 (100%), PGM3 (100%), PHC1 (100%), PHGDH (100%), PHKA1 (100%), PHOX2B (100%), PHYH (100%), PIBF1 (100%), PIEZO2 (100%), PIGA (100%), PIGG (100%), PIGN (100%), PIGQ (100%), PIK3CA (100%), PIK3R5 (100%), PINK1 (100%), PLA2G6 (100%), PLAA (100%), PLCB1 (100%), PLD3 (100%), PLEKHG2 (100%), PLEKHG4 (100%), PLP1 (100%), PLXND1 (100%), PMM2 (100%), PMP22 (100%), PMPCA (100%), PMS1 (100%), PNKD (100%), PNKP (100%), PNP (100%), PNPLA6 (100%), PNPLA8 (100%), PNPT1 (100%), PODXL (100%), POLA1 (100%), POLG (100%), POLG2 (100%), POLR1C (100%), POLR1D (100%), POLR3A (100%), POLR3B (100%), POMGNT1 (100%), POMGNT2 (100%), POMK (100%), POMT1 (100%), POMT2 (100%), PON1 (100%), PON2 (100%), PON3 (100%), POU1F1 (100%), POU4F1 (86.02%), PPARGC1A (100%), PPM1K (100%), PPP1R15B (100%), PPT1 (100%), PQBP1 (100%), PRDM8 (100%), PRF1 (100%), PRICKLE1 (100%), PRKCG (100%), PRKRA (100%), PRNP (100%), PROK2 (100%), PROKR2 (100%), PROP1 (100%), PRPH (100%), PRPS1 (100%), PRRT2 (100%), PRSS12 (100%), PRTN3 (100%), PRX (100%), PSAP (100%), PSAT1 (100%), PSEN1 (100%), PSEN2 (100%), PSMC3IP (100%), PTCH1 (100%), PTCH2 (100%), PTCHD1 (100%), PTPN22 (100%), PTRH2 (100%), PTS (100%), PUM1 (100%), PURA (100%), PYCR2 (100%), QARS (100%), QDPR (100%), QKI (100%), RAB11B (100%), RAB12 (100%), RAB18 (100%), RAB27A (100%), RAB39B (100%), RAB3GAP1 (100%), RAB3GAP2 (100%), RAD50 (100%), RAD51 (100%), RARS (100%), RARS2 (100%), RASA1 (100%), RAX (100%), RBFOX1 (100%), RBM10 (100%), RBPJ (100%), RD3 (100%), RDH12 (100%), REEP1 (100%), REEP2 (100%), RELN (100%), REPS1 (100%), RERE (99.07%), REV3L (100%), RFC2 (100%), RFT1 (100%), RNASEH2A (100%), RNASEH2B (100%), RNASEH2C (100%), RNASET2 (100%), RNF125 (100%), RNF168 (100%), RNF170 (100%), RNF216 (100%), ROGDI (100%), RORA (100%), RPE65 (100%), RPGRIP1 (100%), RPGRIP1L (96.5%), RPIA (100%), RPS6KA3 (100%), RRM2B (100%), RSPH4A (100%), RTEL1 (100%), RTN2 (100%), RTN4IP1 (100%), RTTN (100%), SACS (100%), SAMD9 (100%), SAMD9L (100%), SAMHD1 (100%), SARS2 (100%), SASS6 (100%), SATB2 (100%), SCARB2 (100%), SCN1A (100%), SCN1B (100%), SCN2A (100%), SCN4A (100%), SCN8A (100%), SCN9A (100%), SCO1 (100%), SCO2 (100%), SCP2 (100%), SCYL1 (100%), SDCCAG8 (100%), SDHA (100%), SDHAF1 (100%), SDHB (100%), SDHC (100%), SDHD (100%), SEC23B (100%), SEMA3A (100%), SEPSECS (100%), SERAC1 (100%), SETBP1 (99.5%), SETD2 (100%), SETX (100%), SFXN4 (100%), SGCE (100%), SHANK3 (99.79%), SHH (100%), SIGMAR1 (100%), SIK1 (100%), SIL1 (100%), SIX3 (100%), SIX6 (100%), SKI (100%), SLC12A3 (100%), SLC12A5 (100%), SLC12A6 (100%), SLC13A5 (100%), SLC16A2 (100%), SLC17A5 (100%), SLC18A2 (100%), SLC18A3 (100%), SLC19A2 (100%), SLC19A3 (100%), SLC1A2 (100%), SLC1A3 (100%), SLC1A4 (100%), SLC20A2 (100%), SLC25A1 (100%), SLC25A12 (100%), SLC25A15 (100%), SLC25A22 (100%), SLC25A32 (100%), SLC25A4 (100%), SLC25A46 (100%), SLC26A4 (100%), SLC2A1 (100%), SLC2A3 (100%), SLC30A10 (100%), SLC30A9 (100%), SLC33A1 (100%), SLC39A14 (100%), SLC39A4 (100%), SLC46A1 (99.93%), SLC52A2 (100%), SLC52A3 (100%), SLC5A7 (100%), SLC6A1 (100%), SLC6A17 (100%), SLC6A19 (100%), SLC6A3 (100%), SLC6A5 (100%), SLC6A8 (100%), SLC9A1 (100%), SLC9A6 (100%), SMAD4 (100%), SMARCA4 (98.03%), SMARCB1 (100%), SMC1A (100%), SMPD1 (100%), SNAI2 (100%), SNAP25 (100%), SNAP29 (100%), SNCA (100%), SNCAIP (100%), SNX14 (100%), SOD1 (100%), SORL1 (100%), SOST (100%), SOX10 (100%), SOX2 (100%), SOX3 (100%), SOX5 (100%), SOX6 (100%), SPAST (100%), SPATA5 (100%), SPATA7 (100%), SPG11 (100%), SPG20 (100%), SPG21 (100%), SPG7 (100%), SPIDR (85.35%), SPR (100%), SPRY4 (100%), SPTAN1 (100%), SPTBN2 (100%), SQSTM1 (100%), SRD5A3 (100%), ST3GAL3 (100%), ST3GAL5 (100%), STAMBP (100%), STAT3 (100%), STAT4 (100%), STIL (100%), STRADA (100%), STUB1 (100%), STX11 (100%), STX16 (100%), STXBP1 (100%), STXBP2 (100%), SUCLA2 (100%), SUCLG1 (100%), SUFU (100%), SUMF1 (100%), SUOX (100%), SURF1 (100%), SUZ12 (100%), SYNE1 (100%), SYNGAP1 (97.26%), SYNJ1 (100%), SYT14 (93.48%), SYT2 (100%), TAC3 (100%), TACO1 (100%), TACR3 (100%), TAF1 (100%), TAF15 (100%), TAF2 (100%), TANGO2 (100%), TARDBP (100%), TAT (100%), TBC1D20 (100%), TBC1D24 (100%), TBCD (100%), TBCE (100%), TBK1 (100%), TBL2 (100%), TBX18 (100%), TCF4 (100%), TCN2 (100%), TCOF1 (100%), TCTN1 (100%), TCTN2 (100%), TCTN3 (100%), TDP1 (100%), TDP2 (100%), TEAD1 (100%), TECPR2 (100%), TECR (100%), TELO2 (100%), TENM1 (100%), TERT (100%), TFG (100%), TGFB1 (100%), TGFBR2 (100%), TGFBR3 (100%), TGM6 (100%), TH (100%), THAP1 (100%), THG1L (100%), TIMM8A (100%), TIMMDC1 (100%), TINF2 (100%), TK2 (100%), TLR4 (100%), TMEM126B (100%), TMEM138 (100%), TMEM216 (100%), TMEM231 (100%), TMEM237 (100%), TMEM240 (100%), TMEM5 (100%), TMEM67 (100%), TMEM70 (100%), TOE1 (100%), TOMM40 (100%), TOP2A (100%), TOR1A (100%), TOR1AIP1 (100%), TP53RK (100%), TPI1 (100%), TPK1 (100%), TPM2 (100%), TPM3 (100%), TPP1 (100%), TPP2 (100%), TPRKB (100%), TRAF3IP1 (100%), TRAPPC11 (100%), TRAPPC12 (96.62%), TRAPPC9 (100%), TREM2 (100%), TREX1 (100%), TRIM32 (100%), TRMT5 (100%), TRNT1 (100%), TRPC3 (100%), TRPV4 (100%), TSC2 (100%), TSEN15 (100%), TSEN2 (100%), TSEN34 (100%), TSEN54 (100%), TSFM (93.89%), TTBK2 (100%), TTC19 (100%), TTC8 (100%), TTF1 (100%), TTPA (100%), TTR (100%), TUBA1A (100%), TUBB (100%), TUBB2A (100%), TUBB2B (100%), TUBB3 (100%), TUBB4A (100%), TUBG1 (100%), TUFM (100%), TULP1 (100%), TUSC3 (100%), TWIST1 (100%), TXN2 (100%), TYMP (100%), TYROBP (100%), UBA5 (100%), UBAC2 (100%), UBE3A (100%), UBQLN2 (100%), UBR4 (100%), UBTF (100%), UCHL1 (100%), UFC1 (100%), UNC13A (100%), UNC13D (100%), UNC80 (100%), UPB1 (100%), UQCC2 (100%), UQCC3 (100%), UQCRB (100%), UQCRC2 (100%), UQCRQ (100%), UROC1 (100%), UROD (100%), USB1 (100%), USH1C (100%), USH1G (100%), USH2A (100%), USP8 (100%), VAC14 (100%), VAMP1 (100%), VAPB (100%), VARS2 (100%), VCP (100%), VLDLR (100%), VPS11 (100%), VPS13A (100%), VPS13B (100%), VPS13C (100%), VPS16 (100%), VPS35 (100%), VPS37A (100%), VPS53 (100%), VRK1 (100%), VWA3B (100%), WARS2 (100%), WDPCP (100%), WDR11 (100%), WDR19 (100%), WDR45 (100%), WDR45B (100%), WDR48 (100%), WDR62 (100%), WDR73 (100%), WDR81 (100%), WFS1 (100%), WHSC1 (100%), WNK1 (100%), WRAP53 (100%), WWOX (100%), XPA (100%), XPC (100%), XPR1 (100%), XRCC1 (100%), XRCC4 (100%), YME1L1 (100%), YWHAE (100%), ZBTB18 (100%), ZBTB47 (100%), ZC3H14 (100%), ZC4H2 (100%), ZFR (100%), ZFYVE26 (100%), ZFYVE27 (100%), ZNF335 (100%), ZNF423 (100%), ZNF592 (100%). Genes with Unknown Coverage: AHDC1
