# Supplementary material for: Case report: A novel loss-of-function pathogenic variant in the KCNA1 cytoplasmic N-terminus causing carbamazepine-responsive type 1 episodic ataxia
Source: Front Neurol. 2022 Aug 9;13:975849. doi: 10.3389/fneur.2022.975849 (PMC9397541; doi:10.3389/fneur.2022.975849)
Supplement: Supplementary file 2 [file Data_Sheet_2.PDF]

| Sequence variant | KCNA1 Segment | Primary phenotype | Other Clinical Observations |
|---|---|---|---|
| L155P<br>R167M; A170S;<br>V174F; I176R; I177N | N-terminal<br>Lower S1 | EA1<br>EA1 | |
| F184C | Upper S1 | EA1, seizures | |
| C185W | Upper S1 | EA1, hyperthermia | short sleeper |
| T226A; T226M | S2 | EA1 | |
| T226K | S2 | myokymia | |
| T226R | S2 | EA1, epilepsy | sleep, apnea, sleep latency, developmental delay |
| R239S | S2 | EA1 | |
| A242P | S2 | neuromyotonia, seizures | |
| P244H | S2–3 linker | myokymia | |
| F249C | S2–3 linker | EA1 + Hyperthermia | |
| F249I | S2–3 linker | EA1 | |
| FF>F250 | S2–3 linker | EA1 | breathing difficulties during attacks |
| N255D | S3 | hypomagnesemia | |
| N255K | S3 | paroxysmal kinesigenic dyskinesia | |
| I262T; I262M | S3 | EA1 | |
| E283K | S3–4 linker | EA1 | |
| V299I | S4 | EA1, paradoxical myotonic congenita | |
| F303V; L305F; R307C | S4 | EA1 | |
| G311D; G311S; I314T | S4–5 linker | EA1 | |
| L319R | S4–5 linker | paroxysmal kinesigenic dyskinesia, seizures | |
| R324T | S5 | EA1, epilepsy | |
| E325D | S5 | EA1 | |
| L328V | S5 | hypomagnesemia | |
| L329I | S5 | EA1 | |
| S342I | S5 | EA1, seizures | |
| V368L | S5–S6 pore loop | Epileptic encephalopathy | severe intellectual disability |
| A395S | S6 | EA1 | |
| P403S | S6 proline-valine-proline motif | EA1, epilepsy | very loud breathing at night before age 2, developmental delay, moderate intellectual disability |
| V404I | S6 proline-valine-proline motif | EA1 | mild intellectual disability |
| P405S | S6 proline-valine-proline motif | epileptic encephalopathy | developmental delay, macrocephaly |
| P405L | S6 proline-valine-proline motif | epileptic encephalopathy | pervasive developmental disorder |
| I407M | S6 | EA1 | |
| V408A | S6 | EA1 | |
| V408L | S6 | EA1, seizures | global developmental delay |
| F414C | C Terminus | EA1 | |
| F414S | C Terminus | EA1, epilepsy | |
| R417stop | C Terminus | EA1 | |

**Supplementary Table 1. Genotype-phenotype correlations for human KCNA1 sequence variants.**

Adapted from Paulhus, K., Ammerman, L., and Glasscock, E. (2020). Clinical Spectrum of KCNA1 Mutations: New Insights into Episodic Ataxia and Epilepsy Comorbidity. *Int J Mol Sci* 21(8): 2802.

L155P (the first variant listed) is reported for the first time in the current study.
